# Phenotypic and genetic heterogeneity of tumor tissue and circulating tumor cells in patients with metastatic castrationresistant prostate cancer: a report from the PETRUS prospective study

**DOI:** 10.18632/oncotarget.10396

**Published:** 2016-07-04

**Authors:** Christophe Massard, Marianne Oulhen, Sylvestre Le Moulec, Nathalie Auger, Stéphanie Foulon, Aurélie Abou-Lovergne, Fanny Billiot, Alexander Valent, Virginie Marty, Yohann Loriot, Karim Fizazi, Philippe Vielh, Francoise Farace

**Affiliations:** ^1^ Gustave Roussy, Université Paris-Saclay, Department of Medicine, F-94805, Villejuif, France; ^2^ INSERM, U981 “Identification of Molecular Predictors and New Targets for Cancer Treatment”, F-94805, Villejuif, France; ^3^ Gustave Roussy, Université Paris-Saclay, “Circulating Tumor Cells” Translational Platform, AMMICA CNRS UMS3655 – INSERM US23, F-94805, Villejuif, France; ^4^ Hôpital d'Instruction des Armées du Val de Grâce, Department of Oncology, F-75005, Paris, France; ^5^ Gustave Roussy, Université Paris-Saclay, Department of Biopathology, F-94805, Villejuif, France; ^6^ Gustave Roussy, Université Paris-Saclay, Department of Biostatistics and Epidemiology, F-94805, Villejuif, France; ^7^ Gustave Roussy, Université Paris-Saclay, “Histo Cytopathology” Translational Platform, AMMICA CNRS UMS3655 – INSERM US23, F-94805, Villejuif, France

**Keywords:** prostate cancer, biopsy, circulating tumor cells, androgen receptor, TMPRSS2-ERG translocation

## Abstract

Molecular characterization of cancer samples is hampered by tumor tissue availability in metastatic castration-resistant prostate cancer (mCRPC) patients. We reported the results of prospective PETRUS study of biomarker assessment in paired primary prostatic tumors, metastatic biopsies and circulating tumor cells (CTCs). Among 54 mCRPC patients enrolled, 38 (70%) had biopsies containing more than 50% tumour cells. 28 (52%) patients were analyzed for both tissue samples and CTCs. FISH for *AR*-amplification and *TMPRSS2-ERG* translocation were successful in 54% and 32% in metastatic biopsies and primary tumors, respectively. By comparing CellSearch and filtration (ISET)-enrichment combined to four color immunofluorescent staining, we showed that CellSearch and ISET isolated distinct subpopulations of CTCs: CTCs undergoing epithelial-to-mesenchymal transition, CTC clusters and large CTCs with cytomorphological characteristics but no detectable markers were isolated using ISET. Epithelial CTCs detected by the CellSearch were mostly lost during the ISET-filtration. *AR*-amplification was detected in CellSearch-captured CTCs, but not in ISET-enriched CTCs which harbor exclusively *AR* gain of copies. Eighty-eight percent concordance for *ERG*-rearrangement was observed between metastatic biopsies and CTCs even if additional *ERG*-alteration patterns were detected in ISET-enriched CTCs indicating a higher heterogeneity in CTCs.

Molecular screening of metastatic biopsies is achievable in a multicenter context. Our data indicate that CTCs detected by the CellSearch and the ISET-filtration systems are not only phenotypically but also genetically different. Close attention must be paid to CTC characterization since neither approach tested here fully reflects the tremendous phenotypic and genetic heterogeneity present in CTCs from mCRPC patients.

## INTRODUCTION

The treatment of metastatic castration-resistant prostate cancer (mCRPC) patients has dramatically changed in the last five years thank to the development of active drugs. These include five new agents with proven survival benefit: the chemotherapy agent cabazitaxel, two androgen receptor (AR) pathway inhibitors, abiraterone acetate and enzalutamide, the immunotherapy sipuleucel-T and the radiopharmaceutical radium-223 chloride (Ra-223), along with the bone targeted agent denosumab, which has been shown to reduce morbidity from bone metastases in patients with mCRPC [1–4]. However, not all patients benefit from all these agents and cross-resistance have been reported with some of them [5]. For both the clinical management of individual patients and the development of new therapies, there is an urgent need for new predictive biomarkers that can help oncologists to assess clinical response and guide treatment.

Primary tumor tissue may be poorly representative of a patient's metastatic disease which can arise several years after diagnosis and various therapeutic interventions. Several prospective trials using high-throughput analysis are currently conducted worldwide to characterize the genomic alterations in cancer patients based “on-purpose” metastatic tumor biopsy. In mCRPC, tumor biopsies and particularly bone biopsies are challenging to perform in daily practice, even if it is possible in a well-trained clinical research team infrastructure [6]. The analysis of circulating tumor cells (CTC) offers an attractive non-invasive option to analyze molecular alterations [7–12]. In contrast to tissue biopsies, CTCs are obtained through a noninvasive and easily repeatedly procedure, offering longitudinal information on selected biomarkers during treatment [13, 14]. Moreover CTCs are also likely to be issued from different metastatic sites, and may have the potential to inform on genomic heterogeneity of metastatic deposits [15]. However, the implementation of CTCs has yet to be prospectively established and validated.

Until now the identification and molecular characterization of CTCs have proven difficult due to their rarity and biological heterogeneity. Immunomagnetic capture of EpCAM-positive epithelial CTCs using the semi-automated CellSearch platform has been validated with respect to reproducibility and performance and CellSearch based measurement of CTCs has been cleared by the FDA as an aid to evaluate prognosis in patients with metastatic breast, prostate and colon cancers [16, 17]. In metastatic prostate cancer, large clinical studies have demonstrated that CellSearch-determined CTC counts were the most accurate and independent predictor of overall survival with a better performance than PSA [18–20]. A problem with this method is that CTCs undergoing the epithelial-mesenchymal transition (EMT) may have down-regulated epithelial features including EpCAM expression and can be missed by the CellSearch, a difficulty highlighted by the higher numbers of CTCs recovered using alternative CTC enrichment techniques such as ISET (isolation by size of epithelial tumor cells) filtration [21, 22]. Another challenge remains the feasibility of molecular biomarker analysis using CTCs, mainly due to the low numbers of CTCs captured and the relatively low purity of the cell population, whatever the enrichment method used.

With the emergence of new therapies and the need for biomarker identification in mCRPC, it is becoming increasingly important to evaluate the feasibility of achieving matched molecular analyses in biopsies of metastatic sites and of primary tumors, and CTCs, and assess how the results from CTCs correlate with those of paired tumor samples. Here we report the results of a prospective study which allowed fresh metastatic biopsies, CTCs and archival prostate cancer tissue to be collected, and analyzed for *TMPRSS2-ERG* translocation and *AR*-amplification.

## RESULTS

### Operational feasibility of metastatic tumor biopsies for molecular screening: *AR*-amplification and *ERG-*rearrangement assessment in primary tumors and biopsies of metastases

We first evaluate whether the implementation of biomarkers in clinical practice was feasible by assessing two biomarkers in different sources of cancer samples (primary tumor, fresh metastatic sample and CTCs). Between December 2012 and February 2014, 54 patients were enrolled in the PETRUS study (flow chart in Supplementary Figure 1, Supplementary Table 1). Due to financial constraints, the analyses of *AR*-amplification and *ERG*-rearrangement were performed on CTCs, primary tissue and metastatic biopsy, when available, in 28 patients. *AR*-amplification was detected by FISH in tumor samples. Due to the sensitivity of *ERG* FISH to pre-analytical procedures such as overfixation and difficulty of interpretation due to several possible *ERG*-rearrangement patterns, ERG immunohistochemistry (IHC) was also performed in parallel.

Archival samples from primary diagnosis were not available in 7 (25%) of the 28 patients. No tumor cells were found in two patients (7%). When tumor cells were found, the median percentage of tumor cells was 70% (range: 10-90). Due to primary tumor unavailability or absence of tumor cells, FISH for *AR* amplification was feasible in 15/28 (54%) patients, and ERG status by IHC in 19/28 (68%) patients. No amplification of *AR* was detected in primary tumors. Three patients were positive by ERG IHC and four patients by FISH. Divergent results between ERG IHC and FISH were observed in three patients (Table 1).

**Table 1:** Detection of *ERG* and *AR* alterations in tumor samples from mCRPC patients

	Primary tumor						Metastasis						
Patients	Tumoral cells	ERG expression	*ERG* gene	*AR* gene	Origin	Tumoral cells	ERG expression	*ERG* gene	*AR* gene
										Gain	Rearrangement	Gain	Amplification	Gain	Rearrangement	Gain	Amplification
**P1**	50%	0%	NI^c^	NI^c^	32%	0%	Node	NTC^b^	NTC^b^	NTC^b^	NTC^b^	NTC^b^	NTC^b^
**P2**	80%	0%	NI^c^	NI^c^	NI^c^	NI^c^	Node	60%	0%	54%	0%	42%	0%
**P3**	30%	0%	0%	0%	0%	0%	Node	90%	0%	49%	0%	12%	88%
**P4**	80%	0%	NI^c^	NI^c^	0%	0%	Node	70%	0%	NI^c^	NI^c^	24%	68%
**P5**	90%	0%	NI^c^	NI^c^	0%	0%	Node	95%	0%	0%	0%	0%	100%
**P6**	70%	0%	10%	20%	0%	0%	Bone	70%	0%	NI^c^	NI^c^	0%	0%
**P7**	60%	0%	NI^c^	NI^c^	NI^c^	NI^c^	Peritoneum	NTC^b^	NTC^b^	NTC^b^	NTC^b^	NTC^b^	NTC^b^
**P8**	NTT^a^	NTT^a^	NTT^a^	NTT^a^	NTT^a^	NTT^a^	Node	30%	30%	19%	50%	0%	100%
**P9**	70%	0%	NI^c^	NI^c^	0%	0%	Bone	NTC^b^	NTC^b^	NTC^b^	NTC^b^	NTC^b^	NTC^b^
**P10**	NTT^a^	NTT^a^	NTT^a^	NTT^a^	NTT^a^	NTT^a^	Bone	80%	50%	NI^c^	NI^c^	0%	100%
**P11**	80%	0%	20%	27%	0%	0%	Bone	60%	0%	NI^c^	NI^c^	20%	0%
**P12**	50%	95%	4%	78%	0%	0%	Node	95%	95%	25%	31%	24%	0%
**P13**	80%	0%	12%	32%	0%	0%	Bone	NTC^b^	NTC^b^	NTC^b^	NTC^b^	NTC^b^	NTC^b^
**P14**	NTT^a^	NTT^a^	NTT^a^	NTT^a^	NTT^a^	NTT^a^	Node	80%	0%	35%	0%	0%	100%
**P15**	20%	0%	NI^c^	NI^c^	NI^c^	NI^c^	Bone	NTC^b^	NTC^b^	NTC^b^	NTC^b^	NTC^b^	NTC^b^
**P16**	10%	0%	NI^c^	NI^c^	0%	0%	Bone	70%	0%	NI^c^	NI^c^	50%	0%
**P17**	NTC^b^	NTC^b^	NTC^b^	NTC^b^	NTC^b^	NTC^b^	NTT^a^	NTT^a^	NTT^a^	NTT^a^	NTT^a^	NTT^a^	NTT^a^
**P18**	20%	0%	8%	0%	0%	0%	Node	80%	0%	26%	22%	0%	100%
**P19**	60%	5%	NI^c^	NI^c^	0%	0%	Liver	80%	+	21%	47%	24%	60%
**P20**	NTT^a^	NTT^a^	NTT^a^	NTT^a^	NTT^a^	NTT^a^	Bone	5%	100%	0%	20%	0%	0%
**P21**	70%	0%	0%	0%	0%	0%	Bone	NTC^b^	NTC^b^	NTC^b^	NTC^b^	NTC^b^	NTC^b^
**P22**	90%	0%	NI^c^	NI^c^	NI^c^	NI^c^	Bone	NTC^b^	NTC^b^	NTC^b^	NTC^b^	NTC^b^	NTC^b^
**P23**	NTT^a^	NTT^a^	NTT^a^	NTT^a^	NTT^a^	NTT^a^	Bone	80%	70%	NI^c^	NI^c^	0%	42%
**P24**	80%	60%	NI^c^	NI^c^	0%	0%	Node	70%	80%	0%	28%	0%	88%
**P25**	70%	0%	0%	0%	0%	0%	Node	95%	95%	8%	50%	62%	0%
**P26**	NTC^b^	NTC^b^	NTC^b^	NTC^b^	NTC^b^	NTC^b^	Bone	NTC^b^	NTC^b^	NTC^b^	NTC^b^	NTC^b^	NTC^b^
**P27**	NTT^a^	NTT^a^	NTT^a^	NTT^a^	NTT^a^	NTT^a^	Bone	NTC^b^	NTC^b^	NTC^b^	NTC^b^	NTC^b^	NTC^b^
**P28**	NTT^a^	NTT^a^	NTT^a^	NTT^a^	NTT^a^	NTT^a^	NTT^a^	NTT^a^	NTT^a^	NTT^a^	NTT^a^	NTT^a^	NTT^a^

Abbreviations: *ERG*, ETS-related gene; ETS, erythroblast transformation-specific; *AR*, androgen receptor; mCRPC, metastatic castration-resistant prostate cancer.

a NTT, no tumor tissue available.

b NTC, no tumor cells present in the tumor tissue.

c NI, FISH non interpretable.

In metastasis biopsies no tumor cells were found in 9 patients. When tumor cells were found, the median percentage of tumor cells was 80% (range: 5-95). *AR* amplification and *ERG* status were assessed in 17/28 (61%) patients. Ten patients had an *AR* amplification in the metastatic biopsy, the median percentage of *AR*-amplified cells being of 94% (range, 42-100%). Eight patients were positive by ERG IHC while 7 patients were positive by FISH. One divergent result between ERG IHC and FISH was observed (Table 1).

### Distinct subpopulations of CTCs are isolated in mCRPC patients using the CellSearch and ISET filtration

**Figure 1:**
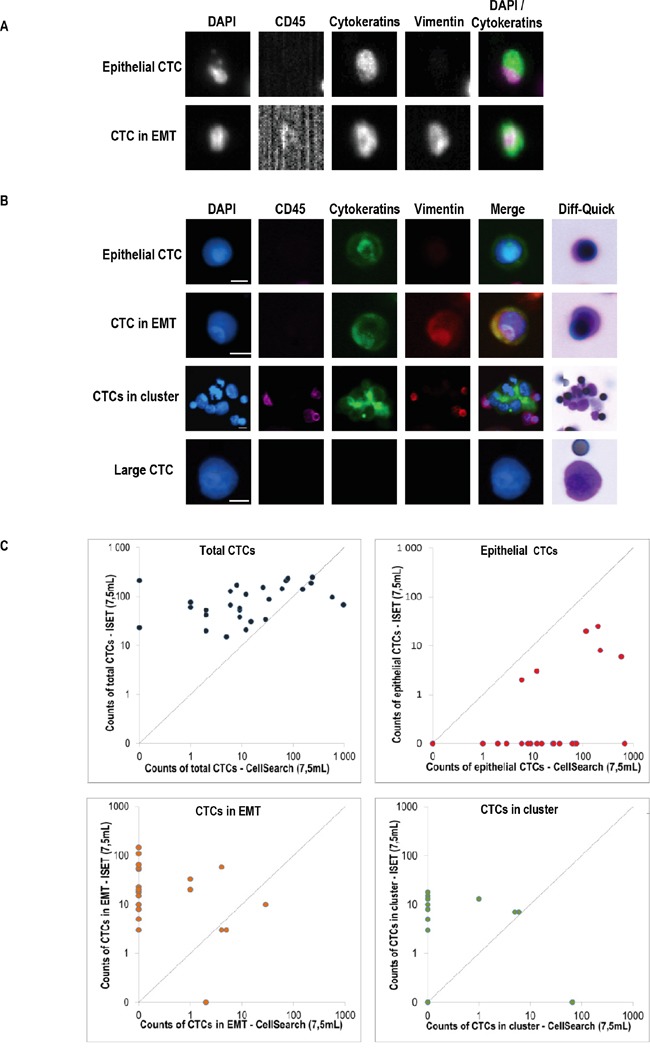
Phenotypic heterogeneity of CTCs isolated by CellSearch and ISET **A.** Representative examples of CTCs detected by CellSearch, **B.** Representative examples of CTCs detected by combining ISET, four-color immunofluorescent staining and imaging on the ARIOL system, **C.** Counts of CTCs by the two methods.

CTCs were analyzed using both the CellSearch and ISET filtration. An anti-vimentin antibody was used in the free channel of the CellSearch to detect EPCAM-positive CTCs expressing vimentin (Figure 1A). ISET-enriched CTCs were tested for EMT marker expression by combining four-color immunofluorescent staining and cytomorphological analysis (Figure 1B) [9, 21]. Table 2 shows the counts of total CTCs, CTC clusters and CTC subpopulations expressing exclusively epithelial markers, or both epithelial and mesenchymal markers (in EMT CTCs) detected using both methods. Median number of total CTCs, CTC clusters, exclusively epithelial CTC and in EMT CTC subpopulations were respectively 11/7.5ml blood (range, 0-973), 0/7.5ml blood (range, 0-66), 11/7.5ml (range, 0-680), 0/7.5ml (range, 0-29) by means of the CellSearch. Median number of total CTCs, CTC clusters, exclusively epithelial CTC and in EMT CTC subpopulations were respectively 72/7.5ml blood (range, 15-249), 5/7.5ml blood (range, 0-18), 0/7.5ml (range, 0-25), 13/7.5ml (range, 0-147) using ISET combined with immunofluorescent staining and cytomorphological analysis. A CTC subset (median, 45/7.5ml; range 4-218) with no detectable epithelial or mesenchymal markers but harboring cytomorphological characteristics of CTCs was consistently detected by ISET (Table 2). Counts of total CTCs, CTC clusters and CTC subpopulations by the two methods were weakly correlated (Figure 1C). These data show that CellSearch and ISET filtration isolated distinct subpopulations of CTCs in mCRPC patients. As expected, the CellSearch detected epithelial CTCs of which few expressed vimentin and formed clusters. In contrast, ISET mostly enriches in CTCs undergoing EMT, CTC clusters and large CTCs with cytomorphological characteristics of tumor cells, but few purely epithelial CTCs.

**Table 2:** CTC subpopulations isolated using CellSearch and ISET according to epithelial and mesenchymal marker expression in mCRPC patients

Patients	CTCs by CellSearch (/7.5mL)				CTCs by ISET (/7.5mL) ^a^				
	Total CTCs	Clusters (CTCs)	Epithelial+	Epithelial+ Vimentin+	Total CTCs	Clusters (CTCs)	Epithelial+	Epithelial+ Vimentin+	Nucleus ≥ 16μm^b^
**P1**	154	6 (9)	116	29	140	7(10)	20	10	100
**P2**	5	0	3	2	15	0	0	0	15
**P3**	237	5(38)	199	0	249	7(15)	25	147	62
**P4**	2	0	1	1	42	5 (5)	0	20	17
**P5**	6	0	6	0	129	15(25)	2	52	50
**P6**	581	0	581	0	97	10(35)	6	18	38
P7	1	0	0	1	76	8(8)	0	33	35
**P8**	0	0	0	0	213	13(15)	0	8	190
**P9**	6	0	6	0	67	8(8)	0	55	4
**P10**	15	0	15	0	31	0	0	23	8
**P11**	225	0	221	4	187	13(13)	8	58	108
**P12**	9	0	9	0	38	0	0	18	20
**P13**	9	0	9	0	53	3(3)	0	10	40
**P14**	9	0	9	0	58	0	0	3	55
**P15**	73	1(3)	70	0	211	13(140)	0	3	68
**P16**	12	0	12	0	111	3(3)	0	20	88
**P17**	79	0	74	5	234	13(13)	0	3	218
**P18**	12	0	12	0	21	0	3	0	18
**P19**	29	0	25	4	34	5(8)	0	3	23
**P20**	2	0	2	0	53	3(8)	0	10	35
**P21**	34	0	34	0	88	3(3)	0	10	75
**P22**	2	0	2	0	20	0	0	5	15
**P23**	973	66(293)	680	0	68	0	0	8	60
**P24**	0	0	0	0	23	0	0	3	20
**P25**	1	0	1	0	60	5(5)	0	15	40
**P26**	26	0	26	0	153	3(3)	0	65	85
**P27**	61	0	61	0	143	18(20)	0	15	108
**P28**	8	0	8	0	170	5(5)	0	110	55

Abbreviations: CTC, circulating tumor cell; ISET, isolation by size of epithelial tumor cells; mCRPC, metastatic castration-resistant prostate cancer.

a CTC subpopulations were characterized by combining a four-color immunofluorescent staining (Cytokeratins 8, 18, 19 and EpCAM in the same chanel, Vimentin, CD45, Hoechst) with cytomorphological staining.

b Large cells with a nucleus ≥ 16 μm, negative for epithelial and mesenchymal markers, include both CTCs and atypical cells identified by an experienced cytopathologist as previously described.

### Detection of *AR*-abnormalities in CTCs

**Figure 2:**
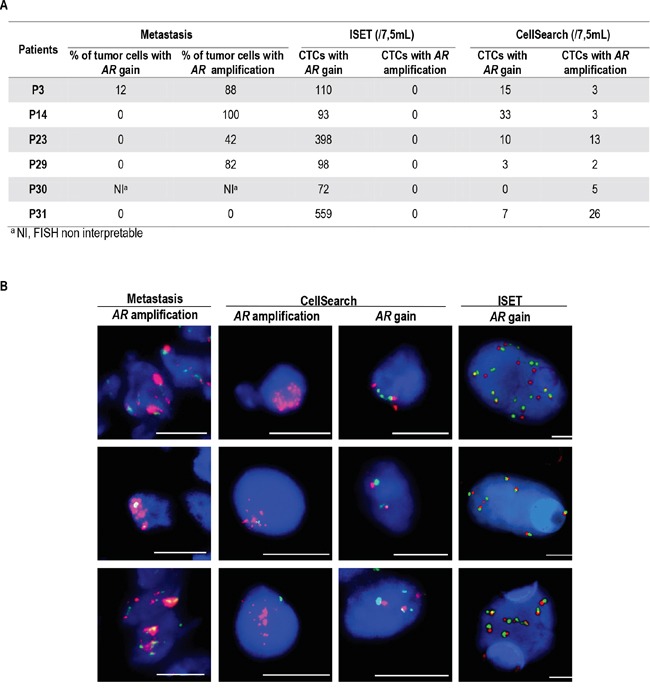
Detection of *AR* amplification and gain of copies in metastasis and CTCs isolated by ISET filtration and CellSearch **A.** Percentages of tumor cells harboring *AR* amplification or gain of copies in metastasis and number of CTCs isolated by ISET filtration and CellSearch harboring *AR* amplification and gain of copies in 6 mCRPC patients, **B.** Examples of FISH patterns of *AR* amplification and gain of copies in metastasis and CTCs isolated by ISET and CellSearch. Scale: bars correspond to 10 μm.

Owing to the higher recovery of CTCs using ISET filtration compared to CellSearch reported in a previous study [21], we next performed FISH on ISET filters using combined immunofluorescent staining (CD45/DAPI) and FA-FISH (filter-adapted FISH) [9, 10]. Gains of *AR* copies were consistently detected in ISET-enriched cells from all patients, but no true amplification of the *AR* was observed (Figure 2), even in patients who harbored *AR*-amplification in the biopsy of the metastasis. These results prompted us to examine *AR*-amplification in CTCs isolated by the CellSearch. In six patients among whom four had *AR*-amplification in the metastatic tissue, CTCs captured by the CellSearch harbored *AR*-amplification (Figure 2A). CTCs harboring gains of *AR* were also captured by the CellSearch but the number of *AR* copies present in individual CTCs was usually lower than that observed in the ISET-enriched fraction. Examples of CTCs isolated by CellSearch and ISET and harboring amplification and gains of *AR* are shown in Figure 2B. These data showed that *AR*-amplified CTCs were missed by ISET-filtration, but could be captured by the CellSearch. Therefore CTCs captured by CellSearch and ISET are not only phenotypically different but also genetically different when considering *AR* gene status.

### Detection of *ERG*-rearrangement in CTCs

**Figure 3:**
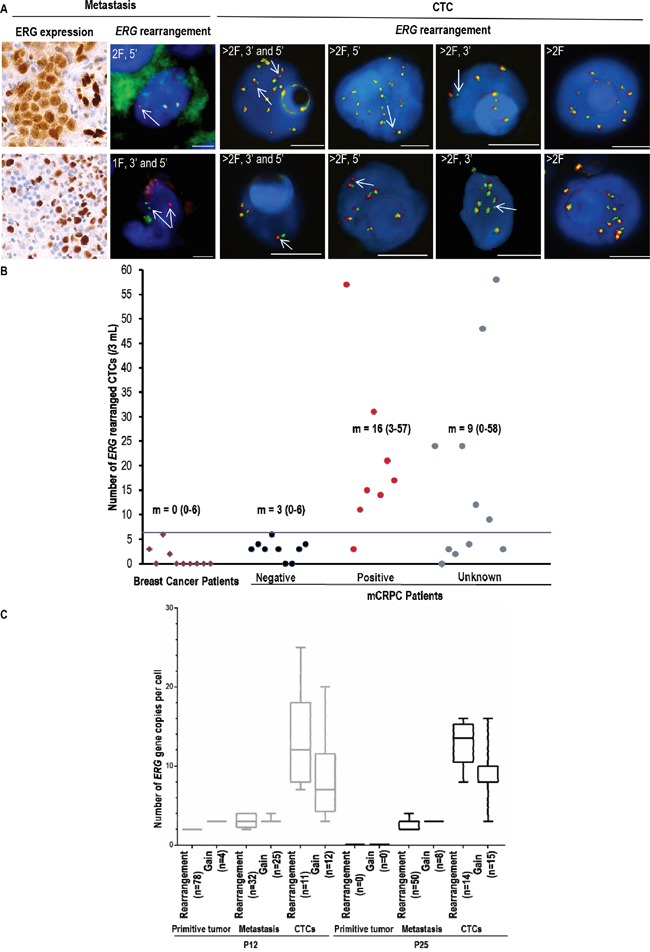
*ERG*-rearrangement detection and molecular heterogeneity of ISET-enriched CTCs bearing *ERG*-alterations in mCRPC patients **A.** Examples of FISH patterns in *ERG*-rearranged cells from metastasis and ISET-enriched CTCs, **B.** Detection of *ERG-*rearranged CTCs in negative breast cancer patients and mCRPC patients according to ERG status by IHC in the metastasis biopsies. m: Median values of *ERG-*rearranged CTCs, **C.** Number of *ERG* copies/cell in *ERG*-rearranged tumor cells and cells bearing only gain of *ERG* copies in primary tumors, metastasis and ISET-enriched CTCs from two mCRPC patients.

*ERG* rearrangement was examined in ISET-enriched CTCs using combined immunofluorescent staining (CD45/DAPI) and FA-FISH (Table 3, Figure 3A). Hybridization background of *ERG* probes was evaluated in a negative cohort of 10 breast cancer patients (Supplementary Table 2, Figure 3B) where the median value of *ERG*-rearranged cells was 0 cell/3ml blood (range 0-6/3ml). The median value of *ERG*-rearranged CTCs was 16/3mL (range, 3-57/3 mL) in the 8 patients exhibiting *ERG*-rearrangement in the metastatic biopsy (Table 3, Figure 3B). The median value of *ERG*-rearranged CTCs was 3/3mL (range, 0-6/3 mL) in the 9 patients without *ERG*-rearrangement in the metastatic biopsy (Table 3, Figure 3B). At a threshold of 7 *ERG*-rearranged CTCs per 3ml blood, *ERG*-rearrangement was detected in CTCs of 7 out of the 8 patients exhibiting *ERG*-rearrangement in the metastatic biopsy while all mCRPC patients negative for *ERG*-rearrangement in the biopsy were negative in CTCs (Table 3, Figure 3B, Supplementary Table 3). The concordance between CTCs and tumors quantified by kappa coefficient was of 88%. At the threshold of 7 *ERG*-rearranged CTCs per 3ml blood, 6 of the 11 mCRPC patients with an undetermined *ERG* status in the metastatic biopsy were found positive for *ERG*-rearrangement in CTCs (Table 3, Figure 3B). In contrast to tumor samples, multiple rearrangement patterns were present in *ERG-*rearranged CTCs regardless of the CTC isolation technique, with gain of native *ERG* far more prevalent (Table 3, Supplementary Table 4, Supplementary Figure 2). In CTCs harboring *ERG*-rearrangement, tumor heterogeneity was assessed by *ERG*-copy number (Supplementary Figure 2). Moreover, in two patients harboring *ERG*-rearrangement in CTCs, tumor heterogeneity seems to be significantly higher in CTCs compared to paired primary tumors and metastasis biopsies (P<0.0001) (Figure 3C).

**Table 3:** Description of *ERG* gene abnormalities in primary tumors, metastasis and ISET-enriched CTCs

		% in primary tumor		% in metastasis	Number of CTCs (/3mL)
	Patients		IHC			≥2F, 3′ and 5′	≥2F, 3′ and 5′, 3′ / ≥2F, 3′	≥2F, 3′ and 5′, 5′ / ≥2F, 5′	Rearranged cells	>2F	Origin	IHC	≥2F, 3′ and 5′	≥2F, 3′ and 5′, 3′ / ≥2F, 3′	≥2F, 3′ and 5′, 5′ / ≥2F, 5′	Rearranged cells	>2F	≥2F, 3′ and 5′	≥2F, 3′ and 5′, 3′ / ≥2F, 3′	≥2F, 3′ and 5′, 5′ / ≥2F, 5′	Rearranged cells	>2F
ERG-Positive	**P8**	NTT^a^	NTT^a^	NTT^a^	NTT^a^	NTT^a^	NTT^a^	Node	30%	12%	19%	19%	50%	19%	12	12	33	57	11
	**P10**	NTT^a^	NTT^a^	NTT^a^	NTT^a^	NTT^a^	NTT^a^	Bone	50%	NI^c^	NI^c^	NI^c^	NI^c^	NI^c^	0	2	1	3	5
	**P12**	95%	17%	48%	13%	78%	4%	Node	95%	12%	0%	19%	31%	25%	4	5	2	11	12
	**P19**	5%	NI^c^	NI^c^	NI^c^	NI^c^	NI^c^	Liver	+ ^d^	24%	0%	23%	47%	21%	7	4	4	15	39
	**P20**	NTT^a^	NTT^a^	NTT^a^	NTT^a^	NTT^a^	NTT^a^	Bone	100%	0%	5%	15%	20%	0%	6	15	0	21	42
	**P23**	NTT^a^	NTT^a^	NTT^a^	NTT^a^	NTT^a^	NTT^a^	Bone	70%	NI^c^	NI^c^	NI^c^	NI^c^	NI^c^	10	13	8	31	78
	**P24**	60%	NI^c^	NI^c^	NI^c^	NI^c^	NI^c^	Node	80%	2%	0%	26%	28%	0%	5	7	5	17	12
	**P25**	0%	0%	0%	0%	0%	0%	Node	95%	20%	6%	24%	50%	8%	3	6	5	14	15
	**P2**	0%	NI^c^	NI^c^	NI^c^	NI^c^	NI^c^	Node	0%	0%	0%	0%	0%	54%	0	3	0	3	48
	**P3**	0%	0%	0%	0%	0%	0%	Node	0%	0%	0%	0%	0%	49%	0	0	4	4	30
	**P4**	0%	NI^c^	NI^c^	NI^c^	NI^c^	NI^c^	Node	0%	NI^c^	NI^c^	NI^c^	NI^c^	NI^c^	3	0	0	3	14
	**P5**	0%	NI^c^	NI^c^	NI^c^	NI^c^	NI^c^	Node	0%	0%	0%	0%	0%	0%	1	3	2	6	60
ERG-Negative	**P6**	0%	0%	14%	6%	20%	10%	Bone	0%	NI^c^	NI^c^	NI^c^	NI^c^	NI^c^	0	3	0	3	81
	**P11**	0%	10%	4%	13%	27%	20%	Bone	0%	NI^c^	NI^c^	NI^c^	NI^c^	NI^c^	0	0	0	0	138
	**P14**	NTT^a^	NTT^a^	NTT^a^	NTT^a^	NTT^a^	NTT^a^	Node	0%	0%	0%	0%	0%	35%	0	0	0	0	54
	**P16**	0%	NI^c^	NI^c^	NI^c^	NI^c^	NI^c^	Bone	0%	NI^c^	NI^c^	NI^c^	NI^c^	NI^c^	0	3	0	3	75
	**P18**	0%	0%	0%	0%	0%	8%	Node	0%	22%	0%	0%	22%	26%	2	0	2	4	33
	**P1**	0%	NI^c^	NI^c^	NI^c^	NI^c^	NI^c^	Node	NTC^b^	NTC^b^	NTC^b^	NTC^b^	NTC^b^	NTC^b^	4	0	5	9	31
	**P7**	0%	NI^c^	NI^c^	NI^c^	NI^c^	NI^c^	Peritoneum	NTC^b^	NTC^b^	NTC^b^	NTC^b^	NTC^b^	NTC^b^	18	3	3	24	54
	**P9**	0%	NI^c^	NI^c^	NI^c^	NI^c^	NI^c^	Bone	NTC^b^	NTC^b^	NTC^b^	NTC^b^	NTC^b^	NTC^b^	0	0	0	0	9
	**P13**	0%	4%	4%	24%	32%	12%	Bone	NTC^b^	NTC^b^	NTC^b^	NTC^b^	NTC^b^	NTC^b^	0	1	0	1	12
	**P15**	0%	NI^c^	NI^c^	NI^c^	NI^c^	NI^c^	Bone	NTC^b^	NTC^b^	NTC^b^	NTC^b^	NTC^b^	NTC^b^	0	0	2	2	63
Unknown ERG status	**P17**	NTC^b^	NTC^b^	NTC^b^	NTC^b^	NTC^b^	NTC^b^	NTT^a^	NTT^a^	NTT^a^	NTT^a^	NTT^a^	NTT^a^	NTT^a^	16	36	6	58	144
	**P21**	0%	0%	0%	0%	0%	0%	Bone	NTC^b^	NTC^b^	NTC^b^	NTC^b^	NTC^b^	NTC^b^	6	18	0	24	99
	**P22**	0%	NI^c^	NI^c^	NI^c^	NI^c^	NI^c^	Bone	NTC^b^	NTC^b^	NTC^b^	NTC^b^	NTC^b^	NTC^b^	2	0	2	4	47
	**P26**	NTC	NTC	NTC	NTC	NTC	NTC	Bone	NTC^b^	NTC^b^	NTC^b^	NTC^b^	NTC^b^	NTC^b^	6	3	3	12	105
	**P27**	NTT^a^	NTT^a^	NTT^a^	NTT^a^	NTT^a^	NTT^a^	Bone	NTC^b^	NTC^b^	NTC^b^	NTC^b^	NTC^b^	NTC^b^	6	6	36	48	72
	**P28**	NTT^a^	NTT^a^	NTT^a^	NTT^a^	NTT^a^	NTT^a^	NTT^a^	NTT^a^	NTT^a^	NTT^a^	NTT^a^	NTT^a^	NTT^a^	3	0	0	3	27

Abbreviations: *ERG*, ETS-related gene; ETS, erythroblast transformation-specific; CTC, circulating tumor cell; ISET, isolation by size of epithelial tumor cells; IHC, immunohistochemistry.

a NTT, no tumor tissue available.

b NTC, no tumor cells present in the tumor tissue.

c NI, FISH non interpretable.

d Positive sample for IHC ERG but difficulty to estimate a percentage.

The red lines correspond to the patients for whom we have the primary tumor, the metastasis and CTCs.

## DISCUSSION

Our study shows that the feasibility to detect biomarkers was questionable in archival primary prostatic tumors because of overfixation or lack of cancer cells. Characterization of the genomic alterations that drives an individual patient's tumor is now critical to select rationally targeted therapies, and it is important to implement prospective molecular triage trials allowing on fresh tumor biopsy analysis. Several prospective trials are ongoing worldwide such as the Dream team project [6, 28], showing that more than 60% of mCRPC have an actionable targets. The present study focused on two key prostate biomarkers (*AR*-amplification, *TMPRSS2-ERG* translocation) useful to classify mCRPC patients into molecular subgroups. Although having currently no direct relevance for a therapeutic decision it is expected that future treatments entering the clinic in mCRPC will be rationally delivered in molecularly selected patient populations according to the presence of these biomarkers. Our results underline the difficulty to assess biomarkers in tumor samples from mCRPC patients and reinforce the need of prospective data concerning bone metastases biopsy and reproducibility of molecular analysis from bone metastases [6].

Observations on the dynamic expression of EpCAM on cancer cells have raised the concern of missing relevant CTCs when using exclusively EpCAM-capture for detection [29]. Our results show that EpCAM-based CTC enrichment by CellSearch and filtration-based CTC enrichment by ISET identifies distinct subpopulations of CTCs in mCRPC patients. Previously we noted a 40% discordance between the results obtained using both systems in patients with prostate cancer [21], but different criteria were used to validate and characterize CTCs at that time. CTCs detected by ISET were identified by a cytopathologist according to morphological criteria while CTCs detected by the CellSearch platform were detected by the intensity of cytokeratin staining and DAPI location. In the present study, we used an immunofluorescent staining to identify ISET-enriched CTCs, further characterized by cytomorphology, and the same antibody combination (anti-EpCAM, pancytokeratins) than that used in the CellSearch. In addition, an anti-vimentin antibody was included in both techniques to detect CTCs undergoing EMT. During EMT cancer cells partially downregulate EpCAM and epithelial features while gradually acquiring mesenchymal characteristics such as vimentin expression. The heterogeneity of CTCs observed here may represent various states of phenotypes and plasticity of cells undergoing EMT, CTCs harboring mesenchymal characteristics being possibly the “more aggressive”. In this sense we recently reported a restrospective study where vimentin expression was analyzed in CTCs detected by the CellSearch technology in 142 samples of 93 patients with advanced prostate cancers. In this report a significant reduction in overall survival was observed in patients with vimentin-positive CTCs compared to those without vimentin-positive CTCs [25]. Otherwise it should be also noted that other mechanisms than EMT can led to EpCAM down-regulation such as hypermethylation of its promoter or intramembranous proteolysis (reviewed in [29]).

Epithelial cells detected by the CellSearch system expressed rarely vimentin but were the only cells where *AR*-amplification could be identified. By contrast, ISET-enriched CTCs were rarely positive for epithelial markers, but highly enriched in cells in clusters, in cells undergoing EMT as well as in large cells lacking any epithelial or EMT markers, and none of them showed *AR*-amplification (only the large cells showed gains of *AR* copy numbers). Hence, *AR*-amplified CTCs are likely exclusively epithelial cells detected by the CellSearch platform, lost during filtration using the ISET system, possibly because of their smaller size. These data indicate that CTCs detected by the CellSearch and the ISET platforms are not only phenotypically but also genetically different, and highlight the potential heterogeneity of CTCs which cannot be covered by a unique CTC isolation technique. These results highlight the need to validate prospectively a CTC platform to assess potential emerging biomarker to guide targeting therapies in mCRPC.

Regardless of the CTC isolation technique, multiple rearrangement patterns were observed in *ERG*-rearranged CTCs, associated with gain of *wild-type ERG* copies far more prevalent than in the tumor samples including metastatic sites. Overexpression of ETS gene has been implicated in cancer progression, and recurrent ETS gene fusions has been found in 50-70% of prostate cancer [30]. Moreover, a recent large study suggested that *ERG* status is associated with worse outcome in mCRPC patients, though these patients derived the greatest benefit from abiraterone [31]. In our study, at a threshold of 7 *ERG*-rearranged CTCs per 3ml blood, *ERG*-rearrangement was detected in CTCs from 7/8 patients positive in metastasis biopsies while the number of *ERG*-rearranged CTCs was lower in all mCRPC patients negative in biopsies of metastases. In ten patients with breast cancer tested by *ERG* FISH to evaluate *ERG* hybridization background in CTCs, numbers of *ERG*-rearranged CTCs were found to be below this threshold. These results show a reasonable concordance between metastatic biopsies and CTCs in our mCRPC patients even if a high level of heterogeneity of *ERG*-abnormalities is observed in CTCs which is much greater than that present in the tumor samples.

We also observed that blood from mCRPC patients is enriched in hyperploid CTCs bearing multiple *ERG* or *AR* abnormalities, potentially representing heterogeneity. CTCs harboring complex cancer genomes have previously been described in peripheral blood [32–35]. Recent studies have demonstrated that multiple subpopulations of CTCs are able to transit between distant sites, seeding between metastases and primary tumors, and thereby supplying the heterogeneous composition of metastases [36, 37]. Such heterogeneous CTCs will acquire an increased probability of molecular pathway alterations by which tumors acquire resistance and the potential to evolve when challenged by treatment. This hypothesis needs to be validated prospectively for example in mCRPC patients resistant to abiraterone or enzalutamide.

In conclusion, metastatic tumor biopsies need to be implemented in molecular screening prospective trials in order to try to develop precision medicine strategies in mCRPC patients. Since non-invasive methods such as CTCs may be proposed in the very next future as one of the main tool for assessing molecular biomarkers, we want to emphasize that, depending on the technique used for isolating CTCs, the data obtained may be dramatically different. Neither the CellSearch nor the ISET approach tested here fully reflect the phenotypic and genotypic heterogeneity observed in CTCs from mCRPC patients and both techniques are complementary for exploring the vast heterogeneity of CTCs in these patients.

## PATIENTS AND METHODS

### Patients

Fifty-five patients with progressive mCRPC, a serum testosterone concentration <0.50 ng/ml (<1.7 nmol/l) and an Eastern Cooperative Oncology Group performance status of 0–2, were included before new therapy initiation in the prospective PETRUS study (NCT01786031). Initial paraffin-embedded prostate cancer specimens were collected when available. Biopsies of metastatic lesions were performed when technically feasible and blood samples were collected before treatment. Biological material collection was approved by an independent ethics committee, and the study was conducted according to the principles of the Declaration of Helsinki, Good Clinical Practice, and applicable regulatory requirements. Ten breast cancer samples were used from the IDRCB2008-A00585-50 study as negative controls. Signed informed consent was obtained from all patients.

### Fluorescent *In situ* hybridization on tumor tissue

The Vysis *AR* amplification Probe Kit consists of two probes: LSI Androgen Receptor Gene (Xq12, Spectrum Orange probe) and CEP X (DXZ1, Spectrum Green probe). *AR* amplification is defined as a ratio of 4:1 between the number of *AR* gene and centromere copies in at least 10% of tumor cells. *AR* gain of copy number is defined by a ratio of 1:1 between the number of *AR* gene and centromere copies with a copy number ≥ 2 in at least 10% of tumor cells. The Kreatech *ERG* Break Apart Rearrangement Probes kit consists of two probes adjacent to the 3′ (green) and 5′ (red) ends of *ERG*. In cells with a *wild-type ERG* status, the overlapping of probes results in a fused (3′5′, yellow) signal. For each biopsy, 3 μm paraffin-embedded tissue sections were deparaffinized and stained with Hematoxylin-Eosin-Safran for tumor tissue examination. Two adjacent biopsy sections were then submitted to dual-color FISH assay using a Dako Pre-treatment Kit (Dako, Glostrup, Denmark). A Vysis *AR* amplification Probe Kit (Abott Molecular Inc., Des Plaines, IL, USA) and a Kreatech *ERG* Break Apart Rearrangement Probe Kit (Kreatech Diagnostics, Netherlands) were used as previously described [23, 24]. The *AR* amplification probe kit consists of two probes: LSI Androgen Receptor Gene (Xq12, Spectrum Orange probe) and CEP X (DXZ1, Spectrum Green probe). A gain of copy number is defined as a 1:1 ratio between the number of *AR* gene copies and centromere probes. *AR* amplification is defined as a 4:1 ratio between number of *AR* gene and centromere copies. The Kreatech *ERG* Break Apart Rearrangement Probes kit consists of two probes adjacent to the 3′ (green) and 5′ (red) ends of *ERG*. In cells with a *wild-type ERG* status, the overlapping of probes results in a fused (3′5′, yellow) signal. *ERG* rearrangement split patterns included the split of 3′ and 5′ probes, and / or an isolated single or amplified 3′ (green) or 5′ (red) signals. Signals were enumerated in at least 50 tumor nuclei and FISH positive cases were defined as those with > 15% of split or isolated signals. Imaging was carried out manually with the epi-fluorescence microscope Eclipse T*i* with a X100 magnification (Nikon Instrument Europe B.V., Surrey, England). FISH patterns were analyzed by a trained experimenter (M.O.) and validated by an experienced cytogeneticist (N.A.).

### Immunohistochemistry on tumor tissue

Immunohistochemistry was performed for ERG (clone 9FY, Biocare Medical, Concord, USA) using a Ventana Benchmark autostainer (Roche Diagnostics, Basel, Switzerland) and standard procedures. Interpretation was performed by a pathologist (P.V.). Imaging was carried out manually with the epi-fluorescence microscope Eclipse T*i* Nikon (Nikon Instrument Europe B.V., Surrey, England) with a X60 magnification.

### CTC detection by CellSearch and enrichment by ISET

Enumeration of CTCs using the CellSearch system (Janssen Diagnostics, Raritan, NJ, USA) was performed according to the manufacturer's protocol [16, 21]. An anti-vimentin antibody conjugated to FITC (V9, Santa Cruz Biotechnology, Dallas, USA) was used in the free channel of the CellSearch as previously [25]. CTC filtration by ISET was performed as previously reported [21, 26].

### Fluorescent *in situ* hybridization on CellSearch-enriched CTCs

Cell suspensions were detached from the magnet walls by gentle aspiration with a micropipette and transferred to an Eppendorf tube. The magnet was rinsed with 300μl PBS 1X and cells centrifuged (1000g, 5min). After supernatant removal, cells were collected in 20 μl and spread onto slides. Slides were dried (45°C, 5 min) and frozen (−20°C) until FISH analysis. FISH was performed as described below for ISET filters.

### Combined immunofluorescent and cytomorphological staining on ISET-enriched CTCs

Enumeration of CTCs was carried out in three spots per patient sample by combining immunofluorescent staining and cytomorphological examination as previously reported [9, 10, detailed in [27]]. Immunofluorescent staining was performed using Hoechst 33342 (Bis-benzimide, B-2261, Sigma Chemical Co., St. Louis, Missouri, USA) (1 μg/ml), Alexa Fluor 488 conjugated anti-EpCAM/CD326 (VU1D9, Novus Biological LLC, Littleton, USA), anti-pancytokeratins (A45B/B3, AS Diagnostik, Hueckeswagen, Germany), anti-Vimentin (V9, Santa Cruz Biotechnology, Dallas, USA) and allophycocyanin (APC)-conjugated anti-CD45 (clone HI30, BD Biosciences, Mississauga, Canada). The anti-pancytokeratin monoclonal antibody was conjugated to Alexa Fluor 488 using Zenon Mouse IgG Labeling Kit (Life Technologies Corp., NY, USA) and anti-vimentin antibody was conjugated to Alexa Fluor 546 using Zenon Mouse IgG Labeling Kit (Life Technologies Corp., NY, USA). Imaging was performed at X20 magnification using an Ariol scanner (Leica Microsystems, Mannheim, Germany). After fluorescent imaging, filters were stained with Diff-Quik set (Siemens Healthcare Diagnostics, Munich, Germany) and scanned again at X20 magnification using the Ariol platform. To identify CTCs, the images obtained after cytological staining were relocated within different CTC phenotypical subpopulations. CTCs were validated by an experienced cytopathologist (P.V.) according to previously reported criteria [21].

### Immunofluorescent staining and filter adapted-fluorescent *In situ* hybridization assay on ISET-enriched CTCs

The two-step method that combined immunofluorescent staining and FA-FISH on filters has been previously reported [9, 10, described in detail in [27]]. FA-FISH were performed with the Vysis *AR* amplification Probe Kit (Abott Molecular Inc., Des Plaines, IL, USA) and the Kreatech *ERG* Break Apart Rearrangement Probes kit (Kreatech Diagnostics, Netherlands). An ISET spot (corresponding to the filtration of one ml of blood) was cut for analysis. Immunofluorescent staining of filters was performed with the monoclonal antibody APC-conjugated anti-CD45 (BD Biosciences, Mississauga, Canada) and DAPI (Life Technologies Corp., NY, USA), then scanned on the Ariol scanner (Leica) and cells were detected at X20 magnification. Single interference filter sets for blue (DAPI), green (FITC) and red (Texas Red) filters were used for FISH scan. DAPI^+^/CD45^−^ cells were precisely located using X63 magnification. Three spots were performed for each patient. All DAPI^+^/CD45^−^ cells were selected. FA-FISH was performed by means of *ERG* and *AR* probe kits used for tumor tissue. All DAPI^+^/CD45^−^ cells present in three ISET spots were analyzed by a trained experimenter (M.O.). FISH patterns were analyzed by a trained experimenter (M.O.) and validated by an experienced cytogeneticist (N.A.). FA-FISH conditions using *ERG* and *AR* probe kits were established using LnCAP and VCAP cell lines (data not shown). As published by Attard *et al*. [7] the absence of *AR* false positive signals was determined by examining the *AR* status of white blood cells (WBC) present in the cell fraction captured by the CellSearch. For all examined patients a normal *AR* status (one *AR* copy) was invariably found in WBC (data not shown). Given the unambiguous nature of *AR* amplification FISH signals the presence of at least two *AR* amplified CTCs was judged sufficient to assess *AR* amplification positivity in CellSearch captured CTCs. The positivity threshold for *ERG*-rearranged CTCs was the value immediately superior to the higher background hybridization value detected in the two negative control cohorts (breast cancer patients (n=10), m CRPC patients without *ERG*-rearrangement in the metastatic biopsy (n=9).

### Statistical analysis

Qualitative data were expressed as numbers and percentages and quantitative data as medians and ranges. Boxplots were used to graphically display the distribution of quantitative data. As a result of the low number of patients and the non-Gaussian distribution of the CTC count, we used the Spearman exact test to study the correlation between the counts of CTCs obtained by CellSearch and ISET. Analyses were performed using SAS 9.3. Concordance between tumor status and levels of *ERG*-rearranged CTCs was determined by the kappa coefficient.

## SUPPLEMENTARY FIGURES AND TABLES



## References

[1] Kantoff PW, Higano CS, Shore ND, Berger ER, Small EJ, Penson DF, Redfern CH, Ferrari AC, Dreicer R, Sims RB, Xu Y, Frohlich MW, Schellhammer PF, Investigators IS (2010). Sipuleucel-T immunotherapy for castration-resistant prostate cancer. The New England journal of medicine.

[2] Parker C, Nilsson S, Heinrich D, Helle SI, O'Sullivan JM, Fossa SD, Chodacki A, Wiechno P, Logue J, Seke M, Widmark A, Johannessen DC, Hoskin P (2013). Alpha emitter radium-223 and survival in metastatic prostate cancer. The New England journal of medicine.

[3] Ryan CJ, Smith MR, de Bono JS, Molina A, Logothetis CJ, de Souza P, Fizazi K, Mainwaring P, Piulats JM, Ng S, Carles J, Mulders PF, Basch E (2013). Abiraterone in metastatic prostate cancer without previous chemotherapy. The New England journal of medicine.

[4] Scher HI, Fizazi K, Saad F, Taplin ME, Sternberg CN, Miller K, de Wit R, Mulders P, Chi KN, Shore ND, Armstrong AJ, Flaig TW, Flechon A (2012). Increased survival with enzalutamide in prostate cancer after chemotherapy. The New England journal of medicine.

[5] Loriot Y, Bianchini D, Ileana E, Sandhu S, Patrikidou A, Pezaro C, Albiges L, Attard G, Fizazi K, De Bono JS, Massard C (2013). Antitumour activity of abiraterone acetate against metastatic castration-resistant prostate cancer progressing after docetaxel and enzalutamide (MDV3100). Annals of oncology.

[6] Small EJ, Huang J, Youngren J, Sokolov A, Aggarwal RR, Thomas G, True LD, Zhang L, Foye A, Alumkal JJ Characterization of neuroendocrine prostate cancer (NEPC) in patients with metastatic castration resistant prostate cancer (mCRPC) resistant to abiraterone (Abi) or enzalutamide (Enz): Preliminary results from the SU2C/PCF/AACR West Coast Prostate Cancer Dream Team (WCDT).

[7] Attard G, Swennenhuis JF, Olmos D, Reid AH, Vickers E, A'Hern R, Levink R, Coumans F, Moreira J, Riisnaes R, Oommen NB, Hawche G, Jameson C (2009). Characterization of ERG, AR and PTEN gene status in circulating tumor cells from patients with castration-resistant prostate cancer. Cancer research.

[8] Miyamoto DT, Sequist LV, Lee RJ (2014). Circulating tumour cells-monitoring treatment response in prostate cancer. Nature reviews Clinical oncology.

[9] Pailler E, Adam J, Barthelemy A, Oulhen M, Auger N, Valent A, Borget I, Planchard D, Taylor M, Andre F, Soria JC, Vielh P, Besse B, Farace F (2013). Detection of circulating tumor cells harboring a unique ALK rearrangement in ALK-positive non-small-cell lung cancer. Journal of clinical oncology.

[10] Pailler E, Auger N, Lindsay CR, Vielh P, Islas-Morris-Hernandez A, Borget I, Ngo-Camus M, Planchard D, Soria JC, Besse B, Farace F (2015). High level of chromosomal instability in circulating tumor cells of ROS1-rearranged non-small-cell lung cancer. Annals of oncology.

[11] Punnoose EA, Ferraldeschi R, Szafer-Glusman E, Tucker EK, Mohan S, Flohr P, Riisnaes R, Miranda S, Figueiredo I, Rodrigues DN, Omlin A, Pezaro C, Zhu J (2015). PTEN loss in circulating tumour cells correlates with PTEN loss in fresh tumour tissue from castration-resistant prostate cancer patients. British journal of cancer.

[12] Leversha MA, Han J, Asgari Z, Danila DC, Lin O, Gonzalez-Espinoza R, Anand A, Lilja H, Heller G, Fleisher M, Scher HI (2009). Fluorescence in situ hybridization analysis of circulating tumor cells in metastatic prostate cancer. Clinical cancer research.

[13] Alix-Panabieres C, Pantel K (2014). Challenges in circulating tumour cell research. Nature reviews Cancer.

[14] Ross K, Pailler E, Faugeroux V, Taylor M, Oulhen M, Auger N, Planchard D, Soria JC, Lindsay CR, Besse B, Vielh P, Farace F (2015). The potential diagnostic power of circulating tumor cell analysis for non-small-cell lung cancer. Expert review of molecular diagnostics.

[15] Shen MM (2015). Cancer: The complex seeds of metastasis. Nature.

[16] Cristofanilli M, Budd GT, Ellis MJ, Stopeck A, Matera J, Miller MC, Reuben JM, Doyle GV, Allard WJ, Terstappen LW, Hayes DF (2004). Circulating tumor cells, disease progression, and survival in metastatic breast cancer. The New England journal of medicine.

[17] Kraan J, Sleijfer S, Strijbos MH, Ignatiadis M, Peeters D, Pierga JY, Farace F, Riethdorf S, Fehm T, Zorzino L, Tibbe AG, Maestro M, Gisbert-Criado R (2011). External quality assurance of circulating tumor cell enumeration using the CellSearch((R)) system: a feasibility study. Cytometry Part B, Clinical cytometry.

[18] de Bono JS, Scher HI, Montgomery RB, Parker C, Miller MC, Tissing H, Doyle GV, Terstappen LW, Pienta KJ, Raghavan D (2008). Circulating tumor cells predict survival benefit from treatment in metastatic castration-resistant prostate cancer. Clinical cancer research.

[19] Goldkorn A, Ely B, Quinn DI, Tangen CM, Fink LM, Xu T, Twardowski P, Van Veldhuizen PJ, Agarwal N, Carducci MA, Monk JP, Datar RH, Garzotto M (2014). Circulating tumor cell counts are prognostic of overall survival in SWOG S0421: a phase III trial of docetaxel with or without atrasentan for metastatic castration-resistant prostate cancer. Journal of clinical oncology.

[20] Scher HI, Heller G, Molina A, Attard G, Danila DC, Jia X, Peng W, Sandhu SK, Olmos D, Riisnaes R, McCormack R, Burzykowski T, Kheoh T, Fleisher M, Buyse M, de Bono JS (2015). Circulating tumor cell biomarker panel as an individual-level surrogate for survival in metastatic castration-resistant prostate cancer. Journal of clinical oncology.

[21] Farace F, Massard C, Vimond N, Drusch F, Jacques N, Billiot F, Laplanche A, Chauchereau A, Lacroix L, Planchard D, Le Moulec S, Andre F, Fizazi K, Soria JC, Vielh P (2011). A direct comparison of CellSearch and ISET for circulating tumour-cell detection in patients with metastatic carcinomas. British journal of cancer.

[22] Krebs MG, Hou JM, Sloane R, Lancashire L, Priest L, Nonaka D, Ward TH, Backen A, Clack G, Hughes A, Ranson M, Blackhall FH, Dive C (2012). Analysis of circulating tumor cells in patients with non-small cell lung cancer using epithelial marker-dependent and -independent approaches. Journal of thoracic oncology.

[23] Brown RS, Edwards J, Dogan A, Payne H, Harland SJ, Bartlett JM, Masters JR (2002). Amplification of the androgen receptor gene in bone metastases from hormone-refractory prostate cancer. The Journal of pathology.

[24] Tomlins SA, Rhodes DR, Perner S, Dhanasekaran SM, Mehra R, Sun XW, Varambally S, Cao X, Tchinda J, Kuefer R, Lee C, Montie JE, Shah RB, Pienta KJ, Rubin MA, Chinnaiyan AM (2005). Recurrent fusion of TMPRSS2 and ETS transcription factor genes in prostate cancer. Science.

[25] Lindsay CR, Le Moulec S, Billiot F, Loriot Y, Ngo-Camus M, Vielh P, Fizazi K, Massard C, Farace F (2016). Vimentin and Ki67 expression in circulating tumour cells derived from castrate-resistant prostate cancer. BMC cancer.

[26] Lecharpentier A, Vielh P, Perez-Moreno P, Planchard D, Soria JC, Farace F (2011). Detection of circulating tumour cells with a hybrid (epithelial/mesenchymal) phenotype in patients with metastatic non-small cell lung cancer. British journal of cancer.

[27] Pailler E, Oulhen M, Billiot F, Galland A, Auger N, Faugeroux F, Laplace-Builhé C, Besse B, Loriot Y, NgoCamus M, Hemamda M, Lindsay C, Soria JC (2016). Method for Semi-Automated Microscopy of Filtration-Enriched Circulating Tumor Cells. BMC cancer.

[28] Robinson D, Van Allen EM, Wu YM, Schultz N, Lonigro RJ, Mosquera JM, Montgomery B, Taplin ME, Pritchard CC, Attard G, Beltran H, Abida W, Bradley RK (2015). Integrative clinical genomics of advanced prostate cancer. Cell.

[29] Gires O, Stoecklein NH (2014). Dynamic EpCAM expression on circulating and disseminating tumor cells: causes and consequences. Cellular and molecular life sciences.

[30] Tomlins SA, Laxman B, Dhanasekaran SM, Helgeson BE, Cao X, Morris DS, Menon A, Jing X, Cao Q, Han B, Yu J, Wang L, Montie JE (2007). Distinct classes of chromosomal rearrangements create oncogenic ETS gene fusions in prostate cancer. Nature.

[31] Attard G, de Bono JS, Logothetis CJ, Fizazi K, Mukherjee SD, Joshua AM, Schrijvers D, van den Eertwegh AJ, Li W, Molina A, Griffin TW, Kheoh T, Ricci DS (2015). Improvements in Radiographic Progression-Free Survival Stratified by ERG Gene Status in Metastatic Castration-Resistant Prostate Cancer Patients Treated with Abiraterone Acetate. Clinical cancer research.

[32] Heitzer E, Auer M, Gasch C, Pichler M, Ulz P, Hoffmann EM, Lax S, Waldispuehl-Geigl J, Mauermann O, Lackner C, Hofler G, Eisner F, Sill H (2013). Complex tumor genomes inferred from single circulating tumor cells by array-CGH and next-generation sequencing. Cancer research.

[33] Steinert G, Scholch S, Niemietz T, Iwata N, Garcia SA, Behrens B, Voigt A, Kloor M, Benner A, Bork U, Rahbari NN, Buchler MW, Stoecklein NH, Weitz J, Koch M (2014). Immune escape and survival mechanisms in circulating tumor cells of colorectal cancer. Cancer research.

[34] Polzer B, Medoro G, Pasch S, Fontana F, Zorzino L, Pestka A, Andergassen U, Meier-Stiegen F, Czyz ZT, Alberter B, Treitschke S, Schamberger T, Sergio M (2014). Molecular profiling of single circulating tumor cells with diagnostic intention. EMBO molecular medicine.

[35] Neves RP, Raba K, Schmidt O, Honisch E, Meier-Stiegen F, Behrens B, Mohlendick B, Fehm T, Neubauer H, Klein CA, Polzer B, Sproll C, Fischer JC, Niederacher D, Stoecklein NH (2014). Genomic high-resolution profiling of single CKpos/CD45neg flow-sorting purified circulating tumor cells from patients with metastatic breast cancer. Clinical chemistry.

[36] Gundem G, Van Loo P, Kremeyer B, Alexandrov LB, Tubio JM, Papaemmanuil E, Brewer DS, Kallio HM, Hognas G, Annala M, Kivinummi K, Goody V, Latimer C (2015). The evolutionary history of lethal metastatic prostate cancer. Nature.

[37] Kim MY, Oskarsson T, Acharyya S, Nguyen DX, Zhang XH, Norton L, Massague J (2009). Tumor self-seeding by circulating cancer cells. Cell.

