# Supplementary material for: Phenotypic and genetic heterogeneity of tumor tissue and circulating tumor cells in patients with metastatic castrationresistant prostate cancer: a report from the PETRUS prospective study
Source: Oncotarget. 2016 Jul 4;7(34):55069–82. doi: 10.18632/oncotarget.10396 (PMC5342402; doi:10.18632/oncotarget.10396)
Supplement: Supplementary file 1 [file oncotarget-07-55069-s001.pdf]

Phenotypic and genetic heterogeneity of tumor tissue and circulating tumor cells in patients with metastatic castration-resistant prostate cancer: a report from the PETRUS prospective study

SUPPLEMENTARY FIGURES AND TABLES

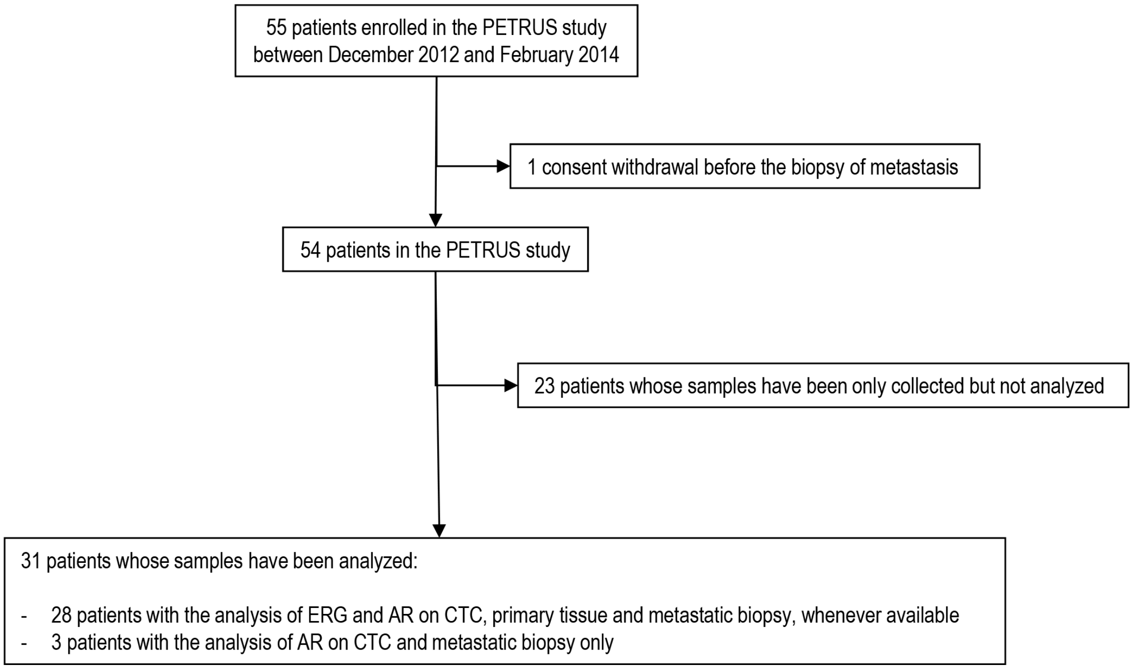

Supplementary Figure S1: Flow chart of tumor tissue and CTC testing in the PETRUS study.

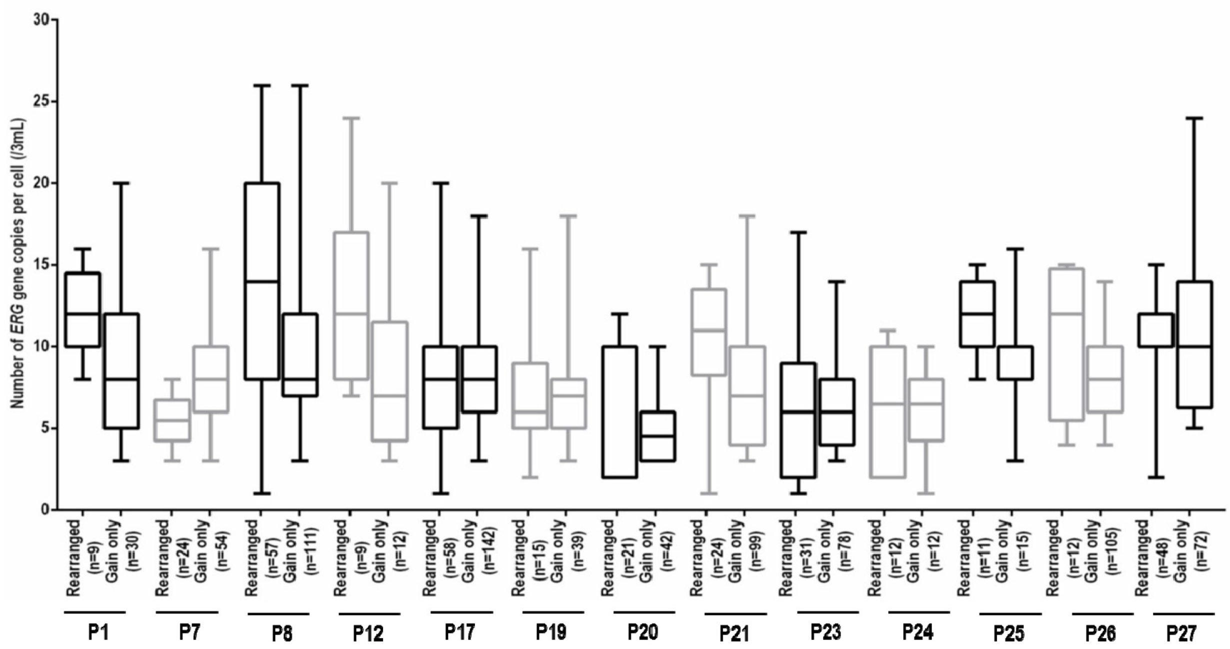

Supplementary Figure S2: Molecular heterogeneity of ISET-enriched CTCs bearing *ERG* alterations. Number of *ERG* copies/cell in *ERG*-rearranged CTCs and CTCs bearing only gain of *ERG* copies in patients considered positive for ISET-enriched CTCs (harboring more than 7 *ERG*-rearranged CTCs per 3 ml of blood). Abbreviations: *ERG*, ETS-related gene; ETS, erythroblast transformation-specific; CTC, circulating tumor cell; ISET, isolation by size of epithelial tumor cells.

Supplementary Table S1: Clinico-biological characteristics of patients

	Total N=54<br>N (%) or median (min ; max)
**Age**	70.6 (51.9 ; 90)
**Total PSA (ng/ml)**	120.1 (0 ; 5783)
**ECOG Performance Status**	
0	16 (29.6%)
1	33 (61.1%)
2	5 (9.3%)
**Patients treated by chemotherapy before the inclusion in this study**	
No	18 (33.3%)
Yes	36 (66.7%)
**Patients treated by Abiraterone before the inclusion in this study**	
No	20 (37%)
Yes	34 (63%)
**Number of metastatic sites$**	2 (1 ; 5)
**Site of the metastatic biopsy**	
Bone	34 (66.7%)
Liver	1 (2.0%)
Nodes	12 (23.5%)
Other tissue	4 (7.8%)
No biopsy	3
**Anti-tumoral treatment received after the inclusion in the study (3MD)**	
Abiraterone	13 (25.5%)
Cabazitaxel	12 (23.5%)
Enzalutamide	9 (17.6%)
Docetaxel	9 (17.6%)
BNIT protocol	1 (2%)
CONET protocol	1 (2%)
Weekly paclitaxel	1 (2%)
Radium 223 dichloride	1 (2%)
Cabozantinib	1 (2%)
Cetuximab	1 (2%)
ODM-201	1 (2%)
Carboplatin-VP16	1 (2%)

$ Among the 5 following sites: bone, liver, node, lung, other tissue
MD: Missing Data

Supplementary Table S2: Description of *ERG* gene alterations in a negative cohort of ten breast cancer patients

Patients	Number of CTCs (/3mL)				
	>2F	≥1F, 3’ and 5’	≥2F, 3’ and 5’, 3’ / ≥2F, 3’	≥2F, 3’ and 5’, 5’ / ≥2F, 5’	Rearranged cells
**P1**	78	0	0	3	3
**P2**	48	0	0	0	0
**P3**	183	3	0	3	6
**P4**	23	0	0	2	2
**P5**	18	0	0	0	0
**P6**	32	0	0	0	0
**P7**	57	0	0	0	0
**P8**	57	0	0	0	0
**P9**	42	0	0	0	0
**P10**	30	0	0	0	0

Abbreviations: *ERG*, ETS-related gene; ETS, erythroblast transformation-specific; CTC, circulating tumor cell.

Supplementary Table S3: Determination of the positivity threshold value for *ERG*-rearranged CTCs

***ERG*-rearranged CTCs**	**ERG status in metastatic samples**		**Total**	
	Positive Patients	Negative Patients		
<7	1	9	10	NPV=90%
≥7	7	0	7	PPV=100%
Total	8	9		
	Sensitivity=85.5%	Specificity=100%		

NPV, Negative Predictive Value; PPV, Positive Predictive Value

Supplementary Table S4: Description of *ERG* gain and rearrangement in CTCs isolated by ISET and CellSearch

Patients	ISET (/3mL)					CellSearch (/3mL)				
	>2F	≥1F, 3’ and 5’	≥2F, 3’ and 5’, 3’ / ≥2F, 3’	≥2F, 3’ and 5’, 5’ / ≥2F, 5’	Rearranged CTCs	>2F	≥1F, 3’ and 5’	≥2F, 3’ and 5’, 3’ / ≥2F, 3’	≥2F, 3’ and 5’, 5’ / ≥2F, 5’	Rearranged CTCs
**P19**	39	7	4	4	15	11	1	7	2	10
**P23**	78	10	13	8	31	17	1	3	0	4
**P11**	138	0	0	0	0	17	0	0	0	0
**P16**	75	0	3	0	3	2	0	4	0	4
**P17**	144	16	36	6	58	2	1	1	0	2
**P21**	99	6	18	0	24	1	1	0	0	1
**P22**	47	2	0	2	4	0	0	1	0	1

Abbreviations: *ERG*, ETS-related gene; ETS, erythroblast transformation-specific; CTC, circulating tumor cell; ISET, isolation by size of epithelial tumor cells.
